# Dietary Supplementation with Fermented *Brassica rapa* L. Stimulates Defecation Accompanying Change in Colonic Bacterial Community Structure

**DOI:** 10.3390/nu13061847

**Published:** 2021-05-28

**Authors:** Sachi Tanaka, Kana Yamamoto, Chisato Hamajima, Fuka Takahashi, Katsunori Endo, Yutaka Uyeno

**Affiliations:** Department of Agriculture, Graduate School of Science and Technology, Shinshu University, Minamiminowa, Nagano 3994598, Japan; k_yamamoto@sk.kameda.co.jp (K.Y.); hamajima.chisato@ma.medience.co.jp (C.H.); ftakahashi@arsoa.co.jp (F.T.); 21hs501a@shinshu-u.ac.jp (K.E.)

**Keywords:** fermented *Brassica rapa* L., bowel function, microbiota, immune function, *Prevotella*

## Abstract

Our previous studies have elucidated that oral administration of *Brassica rapa* L. extract, known as Nozawana in Japan, alters immune responses and gut microbiota composition, increasing the numbers of butyrate-producing bacteria. Therefore, further investigation would help elucidate the mechanism attributable for the changes and health-promoting effects observed after *B rapa* L. extract ingestion. To reveal the modulation effects of fermented *B. rapa* L. on immune function and intestinal bacterial community structure, we conducted an intervention study with healthy volunteers followed by a mouse feeding study. The pilot intervention study was conducted for healthy volunteers aged 40–64 years under the hypothesis that the number of subjects exhibiting any change in gut microbiota in response to fermented *B. rapa* L. consumption may be limited. In total, 20 volunteers consumed 30 g of fermented *B. rapa* L. per day for 4 weeks. The fecal bacterial community composition of the volunteers was characterized using terminal-restriction fragment length polymorphism patterning followed by clustering analysis. To evaluate the detailed changes in the immune responses and the gut bacterial composition, assessed by high-throughput sequencing, we fed healthy mice with freeze-dried, fermented *B. rapa* L. for 2 weeks. The fecal bacterial community composition of the volunteers before the intervention was divided into three clades. Regardless of the clade, the defecation frequency significantly increased during the intervention weeks compared with that before the intervention. However, this clustering detected a specific increase of *Prevotella* in one cluster (low to zero *Prevotella* and high occupation of *Clostridium* at clusters IV and XIVa) post-ingestion. The cytokine production of spleen cells significantly increased due to feeding fermented *B. rapa* L. to the mice. This supplementary in vivo trial provided comparable results to the volunteer study regarding the effects of ingestion of the material given the compositional change complying with that of dietary fiber, particularly in the increase of genera *Prevotella*, *Lachnospira*, and genera in the *Ruminococcaceae* family, and the increase in daily defecation amount during 2 weeks of administration. We conclude that feeding fermented *B. rapa* L. may be responsible for the observed modulation in gut microbiota to increase fiber-degrading bacteria and butyrate-producing bacteria which may be relevant to the improvement in bowel function such as defecation frequency.

## 1. Introduction

Traditional Japanese food is collectively known as washoku, and it has recently been revised given a typical well-balanced diet [1]. Japanese pickles are an important part of the Japanese diet, which supply essential vitamins, minerals, and dietary fibers. *Brassica rapa* L., known as Nozawana in Japan, is a vegetable in the *Brassicaceae* family that contains dietary fiber and vitamin C. It is primarily served as pickled leaves and stalk products (Nozawana-zuke). We verified that the oral administration of *B. rapa* L. extracts to normal mice altered the composition of their gut microbiota and increased the number of butyrate-producing bacteria and the amount of IL-10 production by spleen cells [2]. In a follow-up study, we found that feeding a purified insoluble fraction from fermented *B. rapa* L. affected enteric short-chain fatty acid (SCFA) production as well as immunological responses in the mouse gut [3]. Accordingly, we have successfully compiled information on the gut flora modulating effect of fermented *B. rapa* L. However, thoroughly tracking changes in the community through high-throughput sequencing (HTS) will warrant deeper insight on the mechanism with which the change invokes improvement on animals’ health. Furthermore, the findings we have elucidated so far suggest that the ingestion of fermented *B. rapa* L. might benefit large intestine function in humans, but the effect of regularly consuming this food on human subjects remains unclear.

We therefore aimed at elucidating any changes in gut bacterial structure and concurrent health-promoting effects of consuming fermented *B. rapa* L. We conducted a pilot intervention study in healthy volunteers aged 40–64 years and hypothesized that there may be a limited number of subjects who exhibit any change in gut microbiota in response to fermented *B. rapa* L. consumption. Therefore, we examined the effects of daily ingestion of fermented *B. rapa* on bowel performance, which was determined using defecation frequency and stool consistency scores, and on the microbial ecology and immune function of the gut. Subsequently, an in vivo study was aimed at filling the gap arisen from our previous mouse studies by determining which bacterial groups altered with response to feeding fermented *B. rapa* L. to conventional mice in more detail resolution using HTS methodology. Impact on cytokine production and colonic ecology was also assessed.

## 2. Materials and Methods

### 2.1. Preparation of Fermented B. rapa L. for 2 Experiments

The preparation of fermented *B. rapa* L. generally followed the industrial procedure for commercial products prepared by Marutaka Co., Ltd. (Nagano, Japan). In short, *B. rapa* L. was fermented with NaCl (final 20%) for 6 months. After fermentation, *B. rapa* L. was washed with hot water (60 °C) for 30 min, and then with room temperature water overnight. Before packing, the fermented *B. rapa* L. was sterilized at 110 °C for 15 min. Sterilized bags were filled with 30 g of fermented *B. rapa* L. each. The concentration of NaCl in packed *B. rapa* L. was 0.37%. The content of dietary fiber was ~0.75 g in 30 g of fermented *B. rapa* L.

For the mouse experiment, we used 40 g of the fermented matter, as described above, mixed with 60 mL of distilled water. Next, we sterilized the samples at 121 °C for 20 min, followed by homogenization with an Ace homogenizer AM-3 (Nihon Seiki Seisakusho Ltd., Tokyo, Japan) at 8000 rpm for 3 min. The samples were immediately frozen at −80 °C and subjected to freeze-drying with FDU-1200 freeze-dryer (Eyela, Tokyo, Japan) to produce the freeze-dried material.

### 2.2. Subjects and Study Design of Human Volunteer Study

For this study, we recruited 20 healthy volunteers, 9 female and 11 male, aged 40–64 years. The selection criteria included a body mass index (BMI) in the range of 23–30 kg/m^2^ and a stool frequency of 2–4 times per week, with no recent history of acute or chronic debilitating illness. The BMI (kg/m^2^) was 26.56 ± 0.5 at the end of baseline period and 26.4 ± 0.4 at the end of the consumption period. All subjects were advised not to modify their habitual diet and lifestyle through the study period (including the week that preceded the 4-week intervention). All volunteers signed an informed consent form and agreed to follow the trial guidelines and to provide notification of any noncompliance.

Experiments were performed according to the guidelines of the Helsinki Declaration, and the study protocol was approved by the Ethical Committee for Clinical Experiments of Miura Clinic in Osaka, Japan (approval number S1510). Subjects consumed 30 g of fermented *B. rapa* L. per day during the 4-week intervention based on the ordinal consumption in Japanese cuisine. Several parameters were measured at the beginning and the end of the intervention, such as height, body weight, fat percentage, and blood pressure. Overnight fasting venous blood samples were collected twice, once as a baseline on the day before starting ingestion, and once at the end of the intervention period. These were used for immunological and biochemical analyses. Stool samples collected from all subjects were stored at −80 °C until DNA extraction use.

### 2.3. Serum Biochemistry and Hematology Analyses

The levels of serum lipids, aspartate aminotransferase (AST), alanine aminotransferase (ALT), creatinine, and uric acid were evaluated using reflection spectrometry with an Auto-analyzer 7700 (Hitachi, Tokyo, Japan). Serum glucose was evaluated using a biochemical automatic analyzer, JCA-BM9130 (JEOL, Tokyo, Japan). Red blood cells, hemoglobin, hematocrit, mean corpuscular volume (MCV), platelets, and white blood cells were measured using a hematology analyzer, the XE-2100 (Sysmex, Kobe, Japan).

### 2.4. Preparation of Peripheral Blood Mononuclear Cells

Fasting blood samples were taken from the volunteers, and then peripheral blood mononuclear cells (PBMCs) were prepared from these samples by density gradient separation. The cells were harvested from the interface, washed once, and resuspended in RPMI-1640 medium (Sigma, St. Louis, MO, USA) containing 10% fetal bovine serum (FBS) and 0.75 mM glutamine. Then, the pellet was resuspended in RPMI-1640 medium, and the cell number was adjusted to the required concentration.

### 2.5. Flow Cytometric Analysis

To analyze the proportion of T cells and NK cells, PBMCs were reacted with appropriate concentrations of fluorescence-conjugated monoclonal antibodies to T-cell antigen (CD3) and to NK-cell antigen (CD56). After the addition of the primary antibody and incubation for 15 min at 4 °C, the cells were washed in PBS. Stained cells were analyzed using a flow cytometer.

### 2.6. Natural Killer Cell Activity

Isolated PBMCs were analyzed using the natural killer (NK) cell activity assay previously described by Shida et al. with some modifications [4]. NK cell activity was briefly determined by a ^51^Cr release assay that used ^51^Cr-labeled K562 cells as a target. PBMCs were mixed with target cells in 96-well microculture plates at an effector-to-target (E:T) ratio of 50:1 in an RPMI-1640 medium containing 10% FBS. The plates were incubated at 37 °C in 5% CO_2_. After incubation for 4 h, culture supernatant from each well was collected, and radioactivity in the supernatant was determined by an ARC-370M gamma counter (ALOKA, Co. Ltd., Tokyo, Japan). The lysis percentages were calculated as follows: (experimental point (dpm) − spontaneous release (dpm))/(total release (dpm) − spontaneous release (dpm)) × 100. Values of spontaneous release were <1% of the total release.

### 2.7. Bowel Function Questionnaire

The gastrointestinal symptoms (i.e., defecation frequency and stool consistency) were assessed using a questionnaire, which was filled out daily by each subject. The stool consistency was assessed using the Bristol stool form scale as described previously [5]. We devised a 7-point scale wherein stool samples were scored according to cohesion and surface cracking: 1 = separate hard lumps, like nuts; 2 = sausage-shaped but lumpy; 3 = like a sausage but with cracks on its surface; 4 = like an Italian sausage or snake, smooth and soft; 5 = soft blobs with clear-cut edges; 6 = fluffy pieces with ragged edges, a mushy stool; 7 = watery, no solid pieces, entirely liquid.

### 2.8. Mouse Experiment

The animal experiment was also conducted according to our previous report [3]. The required approval for the experiments was obtained from the Committee for Animal Experiments of Shinshu University (No. 020051). Due to limitations regarding the capacity of the facility and animal management, the data presented in this paper were obtained over the course of 2 feeding trials with identical design (8 animals for each trial, 8 weeks of age for the first trial and 12 weeks of age for the second trial). Non-pregnant, matured C57BL/6 mice were housed in a specific pathogen-free facility. Two diet types were prepared: A standard diet (AIN-93M; 67% carbohydrate, 19% protein, 4% fat, and 4% ash; CON diet) and a diet with fermented *B. rapa* L. (5% of the freeze-dried material with AIN-93M; NZP diet). After a 1-week acclimatization period on a control diet, the animals were split into 2 groups, where 1 group was fed the CON diet and the other was fed NZP diet. Food and water were supplied ad libitum. Mice were fed in 1 cage for 2 weeks, but from 06:00 p.m. on days 7, 10, and 12 to 09:00 a.m. on the following day, mice were separated into individual cages to collect the feces samples. All mice completed the experiment without health impairments. Mice were sacrificed by cervical dislocation, and tissues, including the colon, cecum, and spleen, were collected. Colonic contents (~0.10 g; from the middle part of the colon) were subsampled in 1 mL PBS, mixed thoroughly to equalize the distribution in the buffer, and then immediately frozen at −30 °C. Feces and colonic content samples were used to analyze SCFA with a high-performance liquid chromatography system, as described previously by Tanaka et al. [3].

### 2.9. Measurement of Cytokine Production from Mouse Spleen Cells

Spleens were collected from CON and NZP mice. Spleen cell preparation involved treatment with 0.17 M Tris-HCl buffer (pH 7.65) containing 0.83% NH_4_Cl to deplete the red blood cells. After centrifugation, the cells were resuspended at a concentration of 1 × 10^7^ cells/mL in RPMI-1640 medium containing 10% FBS plus 100 U/mL penicillin G and 100 µg/mL streptomycin (Sigma). The cells were cultured in 96-well flat-bottomed plates in the presence of the LPS (1 μg/mL, Sigma) for 48 h at 37 °C in a 5% CO_2_ atmosphere. Production levels of interferon (IFN)-γ and tumor necrosis factor (TNF)-α in culture supernatants were measured using ELISA kits (eBioscience, San Diego, CA, USA) according to the manufacturer’s instructions.

### 2.10. Microbial Analysis

Microbial analysis of the volunteers’ stool samples was conducted based on a previously described terminal-restriction fragment length polymorphism (T-RFLP) methodology [6]. Stool samples were thawed and used as the source for bacterial genomic DNA extraction from the prokaryotic cells of the suspensions. The extraction was performed using QIAamp DNA Stool Mini Kit (Qiagen, Valencia, CA, USA) according to the manufacturer’s instructions. Solutions of the extracted DNA were stored at −80 °C until use in a T-RFLP analysis. PCR was performed using primers 5′ HEX-labeled 516f and 1510r. The specific conditions for this PCR and the following digestion of the PCR product by 1 restriction enzyme (BslI) were according to those previously described [6]. Each fragment of T-RFLP patterns was classified into its corresponding bacterial group according to descriptions in the literature.

Microbial analysis of mouse gut contents consisted of total bacteria quantification by a quantitative PCR and HTS. The genomic DNA of the microorganisms present in the samples was extracted using the QIAamp DNA Stool Mini Kit and stored at −80 °C until analysis. Total bacteria PCR conditions and primer sequences used in this study are based on those used in a previous study [7]. Primer sets Eub338F (ACTCCTACGGGAGGCAG) and Eub522R (ACGTCRTCCMCNCCTTCCTC), a CFX96 Real-Time PCR detection system (Bio-Rad Inc., Hercules, CA, USA), and an SYBR(R) Premix Ex Taq Kit (Takara Bio Inc., Otsu, Japan) were used. The PCR cycling conditions were initial denaturation at 95 °C for 10 s, followed by 40 cycles at 95 °C for 5 s, and 62 °C for 30 s. Next, we performed melting curve analysis to confirm that the expected PCR products were obtained. Furthermore, the extracted bacterial genomic DNA was subjected to 16S rRNA gene amplicon pyrosequencing. Primer sets 515F (GTGCCAGCMGCCGCGGTAA) and 806R (GGACTACHVHHHTWTCTAAT), a T-100 thermal cycler (Bio-Rad Inc., Hercules, CA, USA), and an Ex Taq Kit (Takara Bio Inc., Otsu, Japan) were used to generate the amplicons. The PCR cycling conditions used for amplification were initial denaturation at 95 °C for 10 s and 25 cycles at 95 °C for 10 s, 57 °C for 30 s, and 62 °C for 30 s for the first PCR, whereas 95 °C for 10 s and 10 cycles at 95 °C for 10 s, 57 °C for 30 s, and 62 °C for 30 s were used for the second PCR. A barcoded amplicon was subjected to paired-end sequencing on an IlluminaMiSeq (Illumina, San Diego, CA, USA). The sequencing results were submitted to the DNA Data Bank of Japan Sequence Read Archive (Accession Number: DRX150611-DRX150626). Post analyses of the sequencing results were carried out after filtering the low-quality reads and trimming the adapters, barcodes, and primers using QIIME. The reads from all the samples were clustered into operational taxonomic units (OTUs) at a 97% sequence similarity level. The data for species-level taxonomy were obtained by filtering the OTU tables containing taxonomic data generated using the Ribosomal Database Project Classifier at the genus level. Next, representative sequences were extracted, and species-level matches within the National Center for Biotechnology Information database were identified using the Basic Local Alignment Search Tool. Further, alpha diversity (α-diversity) was measured using the Shannon index.

### 2.11. Statistical Analysis

For the human volunteer study, we compared T-RFLP fragment patterns of the stool microbiota structure of subjects sampled at 2 timings, respectively, by projecting the numeric data to the Complete-Linkage Clustering method and Euclidean distance. A tree was generated for both timings, and in both the trees, we determined 3 clusters wherein the same subjects were involved. Defecation frequency and stool form were compared based on the cluster basis and the ingestion period by 2-way analysis of variance. The Scheffe method was applied for the post-hoc analysis and in multiple comparisons when a significant difference was determined in any factor. To compare the pre-ingestion (week 0) NK cell and T cell percentages with the NK cell activity with those metrics post-ingestion (week 4) for each subject, a nonparametric Wilcoxon signed-rank test was applied. For the mice study, Student’s t-test was applied to compare data between groups (CON and NZP), unless otherwise stated. We used STATA (version 13.1, Stata Corp, College Station, TX, USA) program for all analyses. *p* < 0.05 was considered statistically significant.

## 3. Results

### 3.1. General Summary of Biochemical Characteristics

All subjects consumed all supplementary items every day during the intervention period. The average body weight tended to decrease after 4 weeks of intervention, but the change was not statistically significant (Table 1). There were no significant effects of fermented *B. rapa* L. on serum glucose, lipids, creatinine, or uric acid levels. However, AST and ALT levels significantly decreased after 4 weeks of supplementation compared with those prior to supplementation (Table 2). Hematological analyses showed that none of the measured hematological parameters changed during the intervention period (Table 3).

### 3.2. Effects on Immune Function of Study Subjects

**Figure 1:**
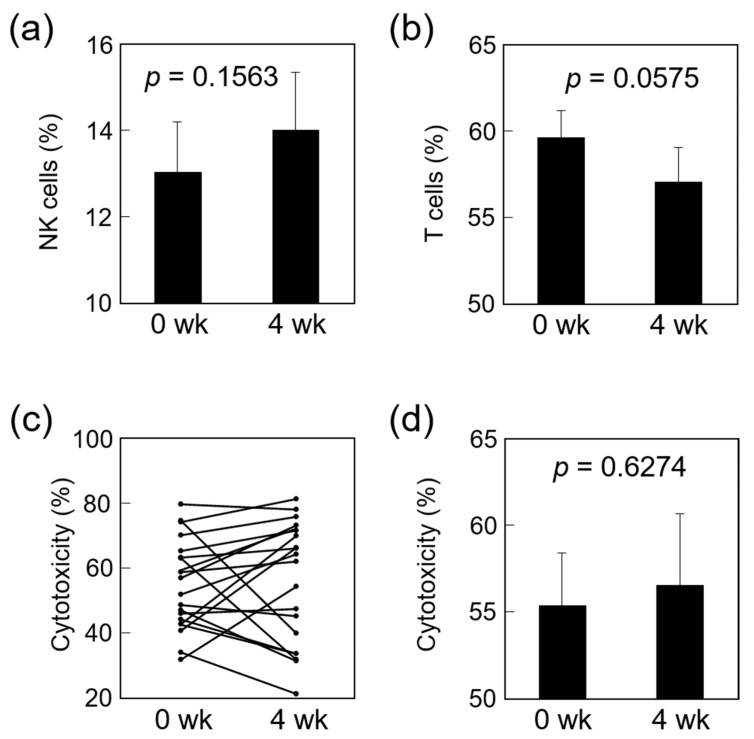
Effect of fermented *B. rapa* L. on immune cells. Each volunteer (*n* = 20) supplemented their diet with fermented *B. rapa* L. for 4 weeks. Percentages of NK cells (**a**) and T cells (**b**) in PBMC from volunteers analyzed using flow cytometry. NK cell activity was measured against K562 target cells at an E/T ratio of 50 by the ^51^Cr-release assay. Changes in NK cell activity in each volunteer (**c**) and mean ± SE (**d**) are shown. Data are mean ± SE. *p*-value was determined by the nonparametric Wilcoxon signed-rank test between week 0 and week 4.

After supplementation with fermented *B. rapa* L., there was a tendency for the percentage of NK cells to be increased (Figure 1a) and the T cells to be decreased (Figure 1b). However, neither of these changes were statistically significant. The NK cell activity of 12 out of the 20 subjects increased after 4 weeks of dietary supplementation with fermented *B. rapa* L. compared with their baseline activity levels (Figure 1c). Moreover, although the average NK cell activity tended to be slightly increased after the 4-week intervention, the change was not statistically significant (Figure 1d). Regarding cytokine production, IFN-γ production was analyzed before and after dietary supplement in these individuals. However, there was no differences in IFN-γ production between before and after dietary supplement (data not shown).

### 3.3. Effects on Stool Microbiota and Bowel Function of Study Subjects

**Figure 2:**
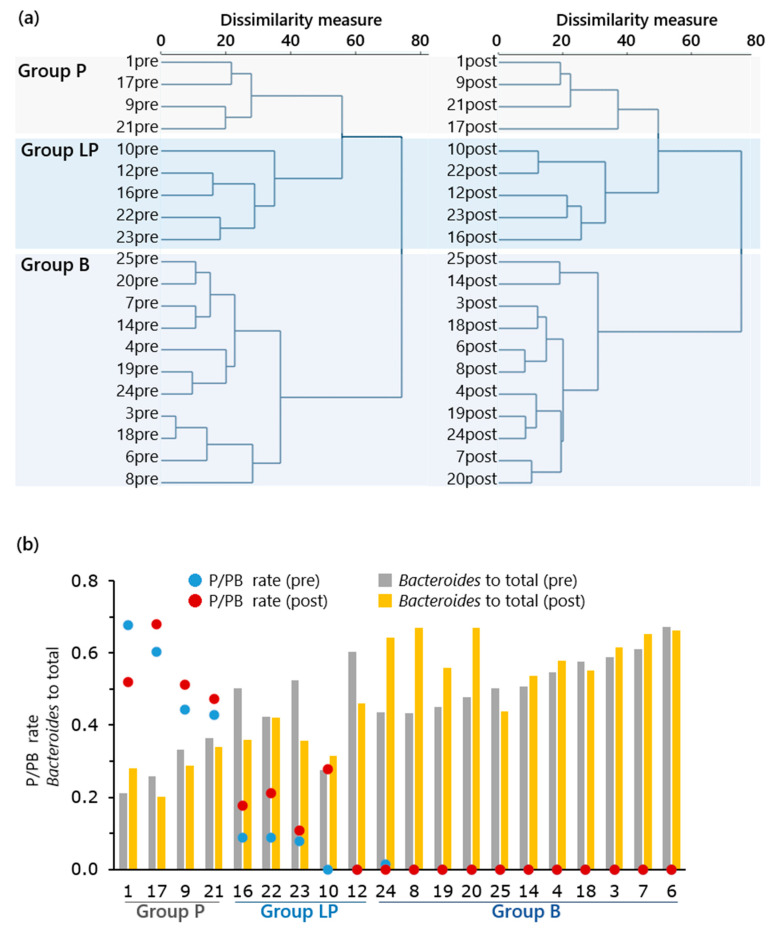
Grouping based on stool microbiota structure. (**a**) Cluster dendrogram of T-RFLP fragment patterns of the stool microbiota structure of subjects sampled twice (pre-ingestion (left) and post-ingestion (right)) separately, based on relative proportions of fragment three distinctive groups. (**b**) Sorting of the subjects based on stool *Prevotella* proportion to the sum of *Prevotella* and *Bacteroides* (P/PB ratio). Two bars for each subject are relative proportions of Bacteroides to total (gray bar, pre-ingestion; yellow bar, post-ingestion). This sorting also well reflected the natures of 3 groups: Group P (high *Prevotella* proportion), group LP (low or zero *Prevotella*, and high *Clostridium* cluster IV and *Clostridium* subcluster XIVa), and group B (low *Clostridium* cluster IV and *Clostridium* subcluster XIVa, and high *Bacteroides*).

We separately compared the T-RFLP fragment patterns of the stool microbiota structure of subjects sampled twice (before and after fermented *B. rapa* L. dietary supplementation) based on the relative proportions of fragments. This processing corresponded to three distinctive groups even during the baseline period: Group P (high *Prevotella* proportion), group LP (low or zero *Prevotella*, and high *Clostridium* cluster IV and *Clostridium* subcluster XIVa), and group B (low *Clostridium* cluster IV and *Clostridium* subcluster XIVa, and high *Bacteroides*), respectively (Figure 2a), as also summarized in Table 4. Another formatting of data, described by the ratio of *Prevotella* to the sum of *Prevotella* and *Bacteroides* (P/PB ratio, with reference to preceding article [8]), well divided subjects’ community profiles into three groups (Figure 2b). The results regarding bowel function are summarized in Table 5. Although it was not significant, defecation frequency before starting ingestion was higher in LP group subjects than the other groups. A significant increase in defecation frequency was found in the later period of the ingestion (week 3 and 4 of intervention) when compared with that during the week before intervention. The mean Bristol stool form scale changed neither in groups nor in the periods.

### 3.4. Mouse Experiment

**Figure 3:**
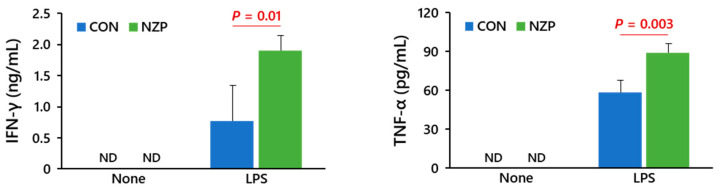
Cytokine production from spleen cells of mice that were fed freeze-dried fermented *B. rapa* L. Spleen cells from mice were cultured in the presence of LPS (1 μg/mL) for 48 h, and then IFN-γ and TNF-α levels were measured using ELISA. The representative data from 2 independent experiments are shown. Data are means ± SD (*n* = 4/group). Significant differences between CON and NZP mice were determined using Student’s *t*-test.

**Figure 4:**
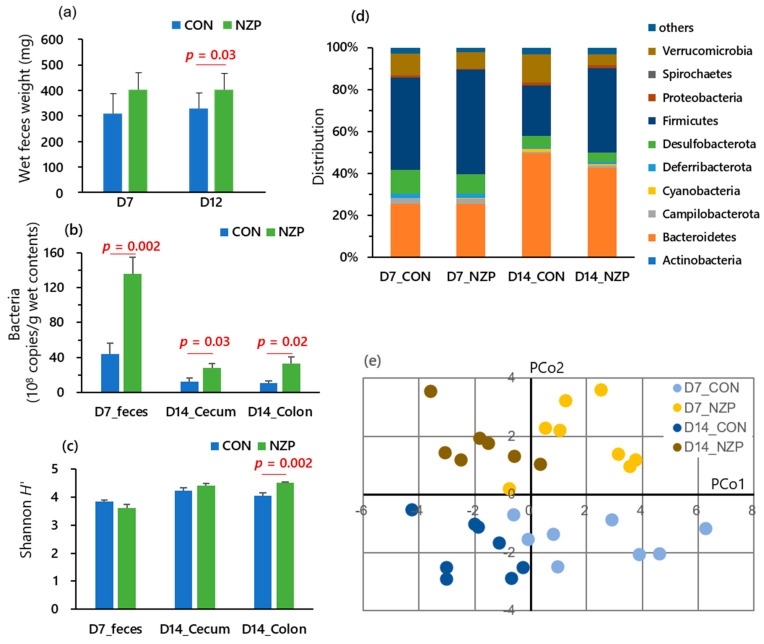
Defecation performance and gut content bacteria characteristics in mice experiment. CON, control group; NZP, treatment group. (**a**) Wet feces weight on the testing day (15 h per collection period). (**b**) Bacterial amounts in three gut content samples (feces sampled at D7, cecum and colon contents sampled at D14). (**c**) Shannon *H* index of bacterial community structure in the three gut content samples. (**d**) Phylum level distribution of the bacteria in each sampling timing (D7 or D14) and each group (CON or NZP). (**e**) Plots of principal coordinate analysis based on genus-level proportions of the bacteria in each group.

To determine the effects of fermented *B. rapa* L. on immune function in mice, cytokine production from spleen cells stimulated with LPS was measured. As a result, in the NZP group, the levels of IFN-γ and TNF-α production significantly increased compared with the CON group (Figure 3). Next, we evaluated the changes in gut microbiota composition. At day 12, fecal weight in the NZP group was higher than that in the CON group, although there was not a significant increase at the 7-day period (Figure 4a). After filtering the low-quality reads of HTS applied, 65,467 to 169,209 effective reads were obtained and further processed by denoising, filtering out chimeras, and removing the archaeal sequences. For all the samples, the median sequence length after preprocessing and before clipping was 283. The total bacterial numbers in the fecal sample on day 7 and colonic sample on day 14 were higher in the NZP group than the CON group (Figure 4b). The Shannon index was also higher in the colonic community of NZP mice than of CON mice (Figure 4c). Comparative analyses revealed a core microbiota across all samples, as the major phylotypes within the community were Bacteroidetes and Firmicutes, followed by Campilobacterota, Deferribacterota, Proteobacteria, and Verrucomicrobia (Figure 4d). Minor differences in the microbial community structures of fecal samples were observed on day 7, but there were significant differences between colonic samples of CON and NZP groups on day 14. Moreover, the distribution of lower-level phylogenetic grouping (family- or genus-level) was different between CON and NZP groups within Bacteroidetes and Firmicutes (Supplementary Figure S1). The result of the principal coordinate analysis based on genus-level proportions well reflected the compositional differences in sample types (7 d feces or 14 d colonic contents) on PCo1 and in treatments (CON or NZP) on PCo2 (Figure 4e). Total SCFA concentration of 7 d feces and butyrate concentration to total of 14 d colonic contents was higher in NZP than CON (Supplementary Figure S2).

## 4. Discussion

The fibrous component in vegetable serves a primarily prebiotic function by increasing the defecation frequency [9,10] and by concurrently modulating the intestinal microbial community of the host to have higher levels of butyrate production [11]. We also reported that the colonic total SCFA level and the butyrate concentration were both higher in mice that were fed a purified insoluble fraction from *B. rapa* L., regardless of the fermentation status [3]. In the volunteers’ trial, we introduced only one intervention (i.e., fermented *B. rapa* L.) to the subject’s lifestyle. We did not impose any further changes on their activities or eating habits and did not measure variables in the washout period. This approach focused on the host’s unique characteristics affecting the degree of microbiota-modulating effects conveyed by *B. rapa* L. ingestion. At the same time, this methodological obstacle was also why we conducted a supplementary experiment using healthy mice to determine in detail the comprehensive structural alteration to the consumption of fermented *B. rapa* L., which helped us link data of recent relevant studies applying HTS formats for community evaluation.

### 4.1. Implications from Results of Volunteer Study

The beneficial effects of fermented vegetables can be attributed to both dietary fiber of the material vegetable and LAB involved in the fermentation process [12,13]. A previous study suggested that fermented *B. rapa* L. possesses an ameliorating effect against pre-symptomatic liver injury [14]. Results from the present study suggest that dietary supplementation with fermented *B. rapa* L. may have implemented another health-promoting function, since the volunteers who had diets supplemented with fermented *B. rapa* L. recorded an improvement in bowel function as assessed using defecation frequency and Bristol stool form scoring.

Regarding the microbial community diversity prior to ingestion, as assumed before conducting this test, the community structure could be divided into three clusters by the T-RFLP profiling approach. Respective clusters could also be characterized by the relative balancing between *Prevotella* and *Bacteroides*, just like *Clostridium* cluster IV and XIVa groups. These are well-recognized groups, including fiber-degrading bacteria. Indeed, the ratio of *Prevotella* to *Prevotella* plus *Bacteroides* was a good biomarker for separating subjects into three groups (Figure 2b) in this study, consistent with preceding observations about comparison regarding the regional difference of fecal community structure in dietary fiber level [8]. Hjorth et al. [15] denoted that individuals with a high *Prevotella*/*Bacteroides* ratio were more susceptible to body weight loss compared with individuals with a low ratio, specifically on a diet rich in fiber and possibly rich in carbohydrates and proteins. The LP group (low or zero *Prevotella* but rich in *Clostridium* clusters IV and XIVa) exhibited a numerical increase in *Prevotella*, except one subject (subject no. 12) who did not exhibit changes in the absence of fecal *Prevotella* in post-ingestion analysis. However, the subject’s flora was rich in *Clostridium* clusters IV and XIVa (41.8% of total), and there was a numeric decrease in *Bacteroides* post-ingestion, unlike the B group subjects. We also conceived that the effects of *B. rapa* L. ingestion was differently exhibited among subjects in each cluster, as high *Prevotella* proportion to *Bacteroides* could help group P subjects perform bowel function more positively. However, the results were not as predicted. The defecation frequency before the consumption differed among clusters, rather than equally increasing regardless of the community composition. In a preceding study, individual differences in gut microbiota caused distinctly different responses to dietary fiber because microbiota dominated by different fiber-utilizing bacteria may have impacted host health by producing different amounts and profiles of SCFAs from the same carbohydrate substrates [16]. A wide range of factors in people’s lives affect their commensal microbial communities and the dynamics of these communities in the larger gut. Therefore, the detailed effects of dietary factors on human gut microbiota and host metabolism and immunity are largely difficult to unveil. Moreover, in the planning of the intervention study, we chose the T-RFLP approach to compare the fecal community structure between pre-consumption and post-consumption periods by a concise mean. Although this approach provides sufficient information regarding the initial purpose, we acknowledge its limitation. The method does not necessarily indicate the proportional change of any other groups than defined taxonomic criteria in the total community, because specific bacteria within each group might have become more prominent following fermented *B. rapa* L. ingestion. A subsequent in vivo test was aimed at addressing this limitation.

### 4.2. Detail Analysis of the Effect of Feeding Fermented B. rapa L. on Gut Bacteria in Mouse

Results from the mouse feeding experiment were consistent with those from the human consumption experiment regarding the defecation measurements (amount or frequency). We also found an increase in SCFA in fecal and colonic contents as well as the increase in total bacterial numbers in these contents. These phenomena could be linked because increased bacteria could be a part of the colonic matter without impairing nutrient digestibility. Increased bacteria were also defecated more frequently due to stimulated colonic movement by the increase in SCFA production [16], probably caused by invoked anaerobic fermentation by fiber-degrading bacteria. Indeed, the population profiling conveyed distinct alteration by fermented *B. rapa* L. ingestion, as well as a significant increase in the genera *Prevotella* and the families *Ruminococcuaceae* and *Lachnospiraceae* (Figure S1). As discussed above, these groups are known to degrade water-soluble and insoluble dietary fiber. This compositional difference is consistent with a recent study [17] showing that mice fed a fiber-free diet showed an increased relative abundance of the families *Verrucomicrobiacaea*, *Porphyromonadaceae*, and *Bacteroidacea*, and a decrease in *Lachnospiraceae*, *Ruminococcaceae*, and *Desulfovibrionaceae*. *Rikenellaceae* was a dominant family of fiber diet-fed mice and the family below the detection limit in fiber-free diet mouse, consistent with our observations. Moreover, in-depth analysis for gut microbiota profile using HTS is in agreement with the volunteer study regarding the ingestion effects on the community structure, which could be shifted to suitably fit to digesting dietary fiber [18].

There are many kinds of immune cells in the intestine that regulate immune function. Besides affecting the local intestinal immune system, these cells can also have a profound influence on systemic immune responses [19]. Immune senescence, defined as age-associated dysfunction of the immune system, is characterized by impaired protective immunity, particularly in cytotoxicity against infectious agents (e.g., cytotoxicity provided by NK cells) [20]. Probiotic microorganisms, including LAB, have been recognized to exert several immunomodulating effects, such as the enhancement of NK cell activity [21] and systemic immunity in older subjects [22]. A previous study found that *Lactobacillus fermentum*, isolated from fermented *B. rapa* L., induced the production of interleukin (IL)-12p40 and IFN-γ frommurine splenocytes [23]. Therefore, imbalances in the gut microbiota, known as dysbiosis, can trigger several immune disorders through the activity of immune cells [24]. The digestive defense system in the gastrointestinal tract includes the mucous layer in the gut, which serves as a physical barrier, and the innate and adaptive immune systems. Many studies in mice and humans have indicated that certain inflammatory diseases are associated with an altered microbiota [25,26]. Certain commensal bacteria appear to preferentially drive regulatory T lymphocyte development [27]. These bacteria can also modulate the host immune system by inducing macrophage activity, which alters the levels of pro- and anti-inflammatory cytokines and increases the NK cell activity [28]. Accordingly, the prebiotic effect of ingesting fermented *B. rapa* L. on immunological function may have occurred via flora modulation. After the ingestion of fermented *B. rapa* L., there were slight changes in the proportions of NK cells and T cells, but these differences were not statistically significant. We previously demonstrated an enhancement of NK activity and T helper type 1 cell (Th1) cytokines, such as IFN-γ and TNF-α, in mice that were orally administered an insoluble fraction of *B. rapa* L. [29]. In the present study, we also confirmed that feeding of fermented *B. rapa* L. significantly increased the production of IFN-γ and TNF-α from spleen cells in mice under changes in microbiota composition. In addition, short-term Chlorella supplementation enhanced NK cell activity, as well as IL-12 and IFN-γ production, and changes in the NK cell activity positively correlated with these Th1 cytokines after the intervention [30]. Given that human NK cell activity can be affected by psychological stress, hormones, aging, and chronic diseases [31], it is not surprising that the average NK cell activity was almost unchanged by the intervention. Variations in NK cell activity may have been attributed to unaccounted factors variable among volunteers rather than the simple ingestion of one type of food. Alternatively, as it has been recognized that *Brassica* vegetables contain various nutrients such as antioxidant vitamins, polyphenols, and minerals, part of the health-promoting effects observed here may have been due to concurrent effects of such active components with dietary fiber of *B. rapa* L. [32].

Although the volunteer experiment presented here was not fully followed to rigorous design, we observed changes in some direct measures, such as defecation frequency and quality and fecal bacteria community composition, which are generally comparable to those induced by the digestion of dietary fiber. The mice feeding experiment well reproduced the changes in defecation amount and in the gut bacterial community structure due to the ingestion of fermented *B. rapa* L. More systematic effects regarding intestinal microbiota-related immune functions to achieve a healthier state of adult humans will also be warranted by a longer and a better-controlled trial of *B. rapa* L. ingestion.

## Figures and Tables

**Table 1:** Baseline characteristics of subjects enrolled in this study.

	0 wk	4 wk	*p*-Value
Age (year)	52.00 ± 1.44		
Male/female (*n*)	11/9		
Weight (kg)	72.88 ± 2.27	72.39 ± 2.25	0.0616
Fat percentage (%)	31.83 ± 1.45	31.80 ± 1.38	0.6291
Systolic blood pressure (mmHg)	133.95 ± 4.58	131.75 ± 4.09	0.3041
Diastolic blood pressure (mmHg)	83.30 ± 3.32	83.60 ± 3.04	1.0000

Data are expressed as the mean ± SE. *p*-values were determined by the nonparametric Wilcoxon signed-rank test. Abbreviation: wk, week; BMI, body mass index; SE, standard error of mean.

**Table 2:** Plasma biochemistry of subjects enrolled in this study.

	0 wk	4 wk	*p*-Value
Glucose (mg/dL)	92.75 ± 5.04	94.4 ± 5.56	0.2463
Total protein (g/dL)	7.44 ± 0.08	7.52 ± 0.08	0.2852
Triglycerides (mg/dL)	124.90 ± 14.76	137.50 ± 13.68	0.3225
Total cholesterol (mg/dL)	229.25 ± 9.53	225.95 ± 8.45	0.6274
HDL cholesterol (mg/dL)	57.95 ± 3.01	57.50 ± 3.96	0.7318
LDL cholesterol (mg/dL)	144.00 ± 8.07	139.75 ± 6.97	0.2861
AST (U/L)	27.60 ± 2.76	23.65 ± 1.97	0.0088
ALT (U/L)	31.45 ± 3.96	26.95 ± 3.43	0.0259
Creatinine (mg/dL)	0.78 ± 0.04	0.77 ± 0.04	0.125
Uric acid (mg/dL)	5.98 ± 0.35	5.95 ± 0.36	0.9679

Data are expressed as the mean ± SE. *p*-values were determined by the nonparametric Wilcoxon signed-rank test. Abbreviations: wk, week; HDL, high density lipoprotein; LDL, low density lipoprotein; AST, aspartate aminotransferase; ALT, alanine aminotransferase; SE, standard error of mean.

**Table 3:** Hematology values of subjects enrolled in this study.

	0 wk	4 wk	*p*-Value
RBCs (10^4^/mL)	480.65 ± 10.92	478.90 ± 9.65	0.7933
Hemoglobin (g/dL)	14.64 ± 0.35	14.55 ± 0.33	0.6044
Hematocrit (%)	43.77 ± 0.81	43.45 ± 0.78	0.5062
MCV (fL)	91.20 ± 1.01	90.85 ± 1.03	0.2527
Platelets (10^4^/mL)	25.79 ± 1.28	26.23 ± 1.40	0.5015
WBCs (10^3^/mL)	5.76 ± 0.33	5.42 ± 0.25	0.1256

Data are expressed as the mean ± SE. *p*-values were determined by the nonparametric Wilcoxon signed-rank test. Abbreviations: wk, week; RBC, red blood cell; MCV, mean corpuscular volume; WBC, white blood cell; SE, standard error of mean.

**Table 4:** Characteristics of the groups divided by the T-RFLP patterns representing fecal bacterial community structure.

Item	Group P	Group LP	Group B
Number of subjects (Subject ID)	4 (1, 9, 17, 21)	5 (10, 12, 16, 22, 23)	11 (3, 4, 6, 7, 8, 14, 18, 19, 20, 24, 25)
Occupation of *Prevotella* ^1^	High (>26%)	Low (<5%) or zero	Zero
Signaturing RFs (Length of the fragment) ^1^	*Clostridium* cluster IX (110) *Prevotella* (317)	*Bacteroides* (366) *Clostridium* subcluster XIVa (494, 940, 990) *Clostridium* cluster IV (749)	*Bacteroides* (469) *Clostridium* cluster XI (338) *Clostridium* subcluster XIVa (754)

^1^ Occupation of *Prevotella* and signaturing RFs in respective groups were judged from the patterns of pre-ingestion fecal samples. Abbreviations: T-RFLP, terminal-restriction fragment length polymorphism; RF, restriction fragment.

**Table 5:** Changes in defecation frequency and stool scaling in response to ingestion of fermented *B. rapa* L., divided by the grouping based on stool bacteria profile.

Item, Period	Group P	Group LP	Group B	Contrast
Defecation frequency (times/day)				
−7 d to 0 d	0.39 ± 0.12	0.51 ± 0.11	0.40 ± 0.18	Group, *p* = 0.12
1 d to 14 d	0.82 ± 0.56	0.83 ± 0.25	0.52 ± 0.27	Period, *p* = 0.02 ^A^
15 d to 28 d	0.77 ± 0.28	0.89 ± 0.27	0.68 ± 0.26	Group × Period, *p* = 0.92
Stool consistency ^1^				
−7 d to 0 d	3.1 ± 0.9	2.8 ± 0.7	2.5 ± 1.2	Group, *p* = 0.27
1 d to 14 d	3.9 ± 0.1	3.1 ± 0.9	3.3 ± 0.8	Period, *p* = 0.21
15 d to 28 d	3.5 ± 0.7	3.2 ± 1.0	3.6 ± 1.0	Group × Period, *p* = 0.87

Data are expressed as the mean ± SD. ^A^ Significant difference was found between the periods −7 d to 0 d and 15 d to 28 d (*p* < 0.05). ^1^ Stool consistency scoring was based on the Bristol stool form scale.

## Data Availability

The sequencing results were submitted to the DNA Data Bank of Japan Sequence Read Archive (Accession Number: DRX150611-DRX150626).

## Supplementary Materials

The following are available online at https://www.mdpi.com/article/10.3390/nu13061847/s1, Figure S1. Lower level (class, family, and genus) distribution of bacteria in colonic contents sampled on day 14. The distribution belonging to Bacteroidetes (a) and Firmicutes (b) were depicted; Figure S2. Total SCFA concentration in gut content samples (a), and relative molar proportions of acetate (b), propionate (c), and butyrate (d).

## References

[1] Oikawa T., Uneyama H. (2016). WASHOKU and health: New approach with dashi/umami in the medical and nutritional health care. Yakugaku zasshi: J. Pharm. Soc. Jpn..

[2] Tanaka S., Yamamoto K., Yamada K., Furuya K., Uyeno Y. (2016). Relationship of enhanced butyrate production by colonic butyrate-producing bacteria to immunomodulatory effects in normal mice fed an insoluble fraction of *Brassica rapa* L.. Appl. Environ. Microbiol..

[3] Tanaka S., Yamamoto K., Hamajima C., Takahashi F., Yamada K., Furuya K., Uyeno Y. (2017). Changes in gut microbial ecology and immunological responses of mice fed the insoluble fraction of *Brassica rapa* L. that was fermented or not. Microbes Environ..

[4] Shida K., Suzuki T., Kiyoshima-Shibata J., Shimada S.-I., Nanno M. (2006). Essential roles of monocytes in stimulating human peripheral blood mononuclear cells with *Lactobacillus casei* to produce cytokines and augment natural killer cell activity. Clin. Vaccine Immunol..

[5] Lewis S.J., Heaton K.W. (1997). Stool form scale as a useful guide to intestinal transit time. Scand. J. Gastroenterol..

[6] Nagashima K., Hisada T., Sato M., Mochizuki J. (2003). Application of new primer-enzyme combinations to terminal restriction fragment length polymorphism profiling of bacterial populations in human feces. Appl. Environ. Microbiol..

[7] Uyeno Y., Akiyama K., Hasunuma T., Yamamoto H., Yokokawa H., Yamaguchi T., Kawashima K., Itoh M., Kushibiki S., Hirako M. (2017). Effects of supplementing an active dry yeast product on rumen microbial community composition and on subsequent rumen fermentation of lactating cows in the mid-to-late lactation period. Anim. Sci. J..

[8] Gorvitovskaia A., Holmes S.P., Huse S.M. (2016). Interpreting *Prevotella* and *Bacteroides* as biomarkers of diet and lifestyle. Microbiome.

[9] Kurasawa S.i., Haack V.S., Marlett J.A. (2000). Plant residue and bacteria as bases for increased stool weight accompanying consumption of higher dietary fiber diets. J. Am. Coll. Nutr..

[10] Yang J., Wang H.-P., Zhou L., Xu C.-F. (2012). Effect of dietary fiber on constipation: A meta analysis. World J. Gastroenterol. WJG.

[11] Uyeno Y., Katayama S., Nakamura S. (2014). Changes in mouse gastrointestinal microbial ecology with ingestion of kale. Benef. Microbes.

[12] Zielińska D., Rzepkowska A., Radawska A., Zieliński K. (2015). In vitro screening of selected probiotic properties of *Lactobacillus* strains isolated from traditional fermented cabbage and cucumber. Curr. Microbiol..

[13] Chiu H.H., Tsai C.C., Hsih H.Y., Tsen H.Y. (2008). Screening from pickled vegetables the potential probiotic strains of lactic acid bacteria able to inhibit the *Salmonella* invasion in mice. J. Appl. Microbiol..

[14] Nyblom H., Berggren U., Balldin J., Olsson R. (2004). High AST/ALT ratio may indicate advanced alcoholic liver disease rather than heavy drinking. Alcohol Alcohol..

[15] Hjorth M.F., Blædel T., Bendtsen L.Q., Lorenzen J.K., Holm J.B., Kiilerich P., Roager H.M., Kristiansen K., Larsen L.H., Astrup A. (2019). *Prevotella*-to-*Bacteroides* ratio predicts body weight and fat loss success on 24-week diets varying in macronutrient composition and dietary fiber: Results from a post-hoc analysis. Int. J. Obes..

[16] Chen T., Long W., Zhang C., Liu S., Zhao L., Hamaker B.R. (2017). Fiber-utilizing capacity varies in *Prevotella*- versus *Bacteroides*-dominated gut microbiota. Sci. Rep..

[17] Fischer F., Romero R., Hellhund A., Linne U., Bertrams W., Pinkenburg O., Eldin H.S., Binder K., Jacob R., Walker A. (2020). Dietary cellulose induces anti-inflammatory immunity and transcriptional programs via maturation of the intestinal microbiota. Gut Microbes.

[18] Kovatcheva-Datchary P., Nilsson A., Akrami R., Lee Y.S., De Vadder F., Arora T., Hallen A., Martens E., Björck I., Bäckhed F. (2015). Dietary Fiber-Induced Improvement in Glucose Metabolism Is Associated with Increased Abundance of Prevotella. Cell Metab..

[19] Mowat A.M., Agace W.W. (2014). Regional specialization within the intestinal immune system. Nat. Rev. Immunol..

[20] Ginaldi L., Loreto M.F., Corsi M.P., Modesti M., De Martinis M. (2001). Immunosenescence and infectious diseases. Microb. Infect..

[21] Takeda K., Okumura K. (2007). Effects of a fermented milk drink containing *Lactobacillus casei* strain Shirota on the human NK-cell activity. J. Nutr..

[22] Moro-García M.A., Alonso-Arias R., Baltadjieva M., Benítez C.F., Barrial M.A.F., Ruisánchez E.D., Santos R.A., Sánchez M.Á., Miján J.S., López-Larrea C. (2013). Oral supplementation with *Lactobacillus delbrueckii* subsp. *bulgaricus* 8481 enhances systemic immunity in elderly subjects. Age.

[23] Kawahara T., Otani H. (2006). Stimulatory effect of lactic acid bacteria from commercially available nozawana-zuke pickle on cytokine expression by mouse spleen cells. Biosci. Biotech. Biochem..

[24] Bouladoux N., Hall J., Grainger J., Dos Santos L., Kann M., Nagarajan V., Verthelyi D., Belkaid Y. (2012). Regulatory role of suppressive motifs from commensal DNA. Mucosal Immunol..

[25] Abraham C., Medzhitov R. (2011). Interactions between the host innate immune system and microbes in inflammatory bowel disease. Gastroenterology.

[26] Delzenne N.M., Neyrinck A.M., Cani P.D. (2013). Gut microbiota and metabolic disorders: How prebiotic can work?. Br. J. Nutr..

[27] Arpaia N., Campbell C., Fan X., Dikiy S., van der Veeken J., Liu H., Cross J.R., Pfeffer K., Coffer P.J., Rudensky A.Y. (2013). Metabolites produced by commensal bacteria promote peripheral regulatory T-cell generation. Nature.

[28] Martins E.M.F., Ramos A.M., Vanzela E.S.L., Stringheta P.C., de Oliveira Pinto C.L., Martins J.M. (2013). Products of vegetable origin: A new alternative for the consumption of probiotic bacteria. Food Res. Int..

[29] Yamamoto K., Furuya K., Yamada K., Takahashi F., Hamajima C., Tanaka S. (2018). Enhancement of natural killer activity and IFN-γ production in an IL-12-dependent manner by a *Brassica rapa* L.. Biosci. Biotech. Biochem..

[30] Kwak J.H., Baek S.H., Woo Y., Han J.K., Kim B.G., Kim O.Y., Lee J.H. (2012). Beneficial immunostimulatory effect of short-term *Chlorella* supplementation: Enhancement of natural killer cell activity and early inflammatory response (randomized, double-blinded, placebo-controlled trial). Nutr. J..

[31] Kiecolt-Glaser J.K., McGuire L., Robles T.F., Glaser R. (2002). Psychoneuroimmunology: Psychological influences on immune function and health. J. Consult. Clin. Psychol..

[32] Aiso I., Inoue H., Seiyama Y., Kuwano T. (2014). Compared with the intake of commercial vegetable juice, the intake of fresh fruit and komatsuna (*Brassica rapa* L. var. *perviridis*) juice mixture reduces serum cholesterol in middle-aged men: A randomized controlled pilot study. Lipids Health Dis..

