# Supplementary material for: Dietary Supplementation with Fermented Brassica rapa L. Stimulates Defecation Accompanying Change in Colonic Bacterial Community Structure
Source: Nutrients. 2021 May 28;13(6):1847. doi: 10.3390/nu13061847 (PMC8227684; doi:10.3390/nu13061847)
Supplement: Supplementary file 1 [file nutrients-13-01847-s001.zip › nutrients-1223449-supplementary.pdf]

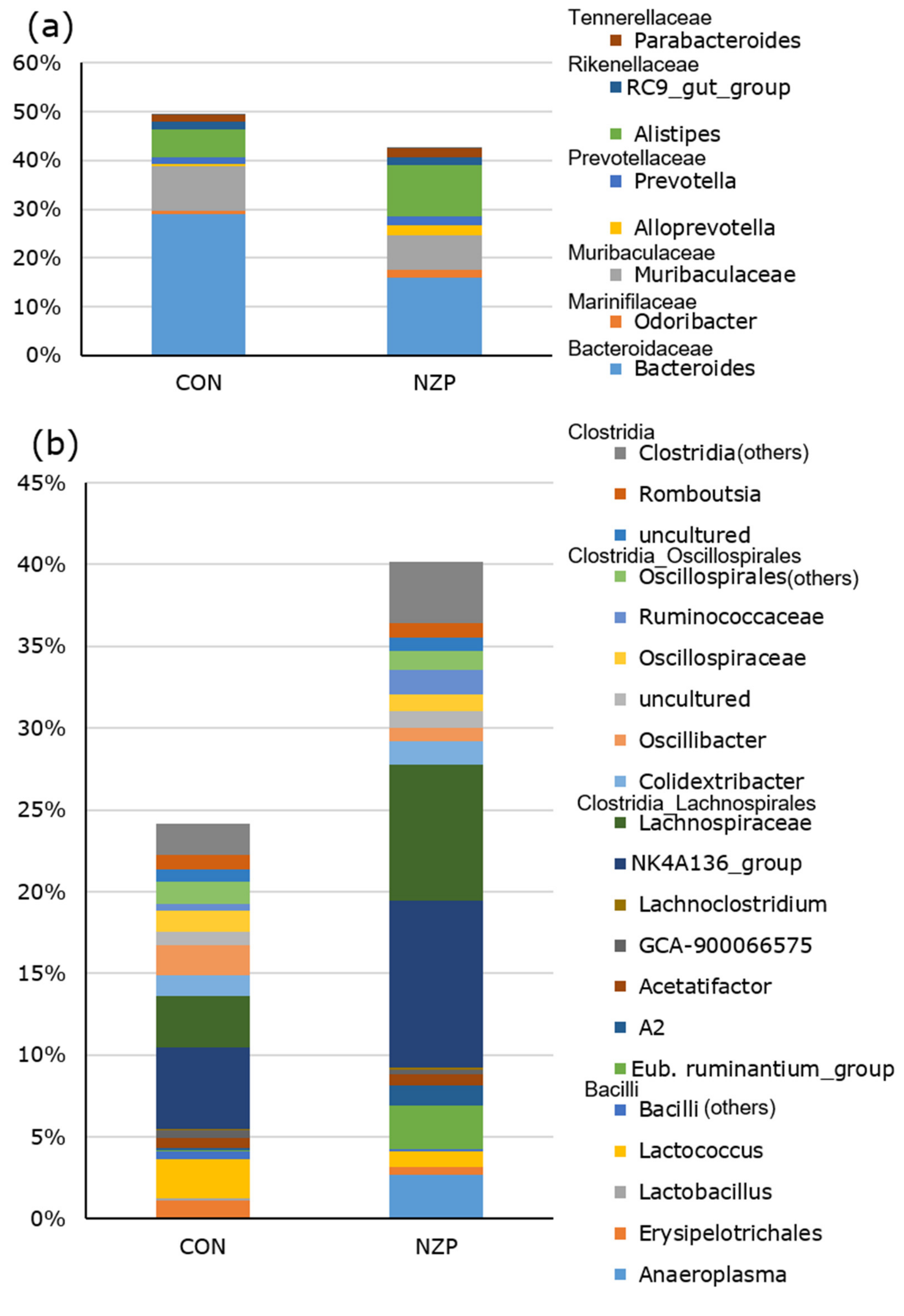

Figure S1. Lower level (class, family, and genus) distribution of bacteria in colonic contents sampled on day 14. The distribution belonging to Bacteroidetes (a) and Firmicutes (b) were depicted.

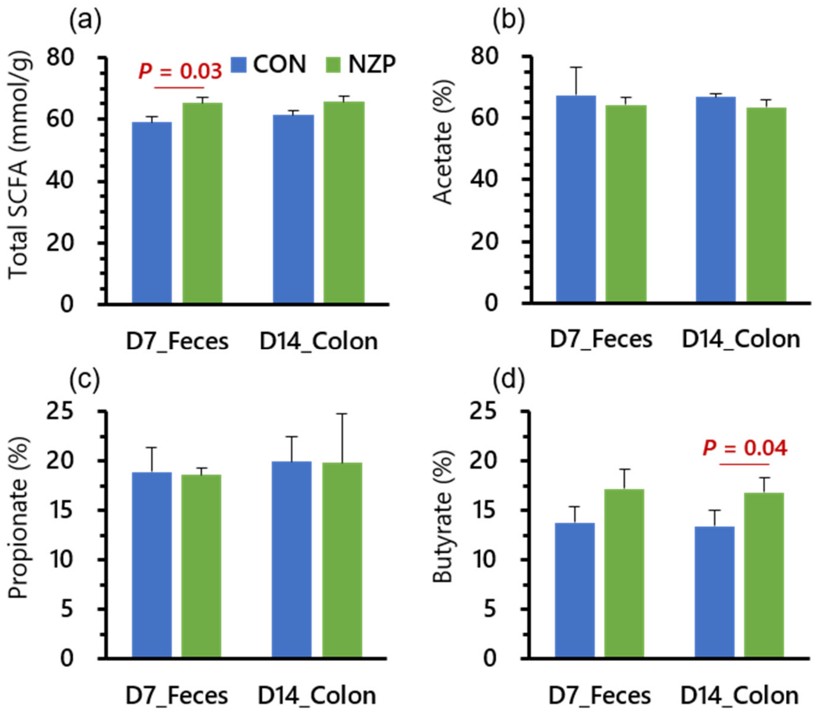

Figure S2. Total SCFA concentration in gut content samples (a), and relative molar proportions of acetate (b), propionate (c), and butyrate (d).
